# Colonisation with pathogenic drug-resistant bacteria and *Clostridioides difficile* among residents of residential care facilities in Cape Town, South Africa: a cross-sectional prevalence study

**DOI:** 10.1186/s13756-019-0643-y

**Published:** 2019-11-19

**Authors:** Jason September, Leon Geffen, Kathryn Manning, Preneshni Naicker, Cheryl Faro, Marc Mendelson, Sean Wasserman

**Affiliations:** 1 Department of Medicine, University of Cape Town, Cape Town, South Africa; 2 Samson Institute for Ageing Research. Institute of Ageing in Africa, University of Cape Town, Cape Town, South Africa; 3 Division of Medical Microbiology, University of Cape Town, Cape Town, South Africa; 4 Division of Infectious Diseases and HIV Medicine, University of Cape Town, Cape Town, South Africa; 5 Wellcome Centre for Infectious Diseases Research in Africa, University of Cape Town, Cape Town, South Africa

**Keywords:** Residential care facility, Antibiotic resistance, *C. difficile*, Colonization, MRSA, ESBL, Infection control, Antibiotic stewardship

## Abstract

**Background:**

Residential care facilities (RCFs) act as reservoirs for multidrug-resistant organisms (MDRO). There are scarce data on colonisation with MDROs in Africa. We aimed to determine the prevalence of MDROs and *C. difficile* and risk factors for carriage amongst residents of RCFs in Cape Town, South Africa.

**Methods:**

We performed a cross-sectional surveillance study at three RCFs. Chromogenic agar was used to screen skin swabs for methicillin-resistant *S. aureus* (MRSA) and stool samples for extended-spectrum beta-lactamase-producing Enterobacteriaceae (ESBL-E). Antigen testing and PCR was used to detect *Clostridiodes difficile*. Risk factors for colonisation were determined with logistic regression.

**Results:**

One hundred fifty-four residents were enrolled, providing 119 stool samples and 152 sets of skin swabs. Twenty-seven (22.7%) stool samples were positive for ESBL-E, and 13 (8.6%) residents had at least one skin swab positive for MRSA. Two (1.6%) stool samples tested positive for *C. difficile*. Poor functional status (OR 1.3 (95% CI, 1.0–1.6)) and incontinence (OR 2.9 (95% CI, 1.2–6.9)) were significant predictors for ESBL-E colonisation. MRSA colonization appeared higher in frail care areas (8/58 v 5/94, *p* = 0.07).

**Conclusions:**

There was a relatively high prevalence of colonisation with MDROs, particularly ESBL-E, but low *C. difficile* carriage, with implications for antibiotic prescribing and infection control practice.

## Background

Antibiotic resistance (ABR) is a global public health crisis undermining the ability to treat bacterial infections. ABR is the inevitable consequence of antibiotic use in human health and the environment and may correlate with antibiotic consumption [1, 2]. The increase in multidrug-resistant organisms (MDRO) has necessitated a change in empiric antibiotic prescribing practices, and patients with healthcare-associated infections, including from residential care facilities (RCFs), are now often treated with second-line broad-spectrum antibiotics [3]. It is therefore critical to risk-stratify patients for infection with MDRO to support optimal antibiotic prescribing.

Colonisation (defined as asymptomatic carriage) with MDROs is a well-established risk factor for infection with the same strain [4, 5], particularly in immunocompromised and elderly populations [6, 7]. RCFs are increasingly recognized as reservoirs for MDROs [4, 8, 9] and colonisation with MDR bacteria has been associated with outbreaks after referral of RCF residents to acute care facilities [10]. Additionally, residents of RCFs in high income countries have high rates of *Clostridioides difficile* (previously *Clostridium difficile*) colonisation [11] and are susceptible to *C. difficile* infection (CDI) because of advanced age and frequent antibiotic use [12].

ABR is common in South African referral hospitals. Up to 70% of *K. pneumoniae* bloodstream isolates are extended-spectrum beta-lactamase (ESBL) producing strains [13], defined as being resistant to beta-lactam antibiotics, including third-generation cephalosporins such as cefotaxime, ceftriaxone, and ceftazidime. Almost a quarter of *Staphylococcus aureus* bloodstream infections at one tertiary academic centre were resistant to cloxacillin (methicillin-resistant *S. aureus*, MRSA) [13]. There are no published data on the prevalence of colonisation with MDROs or *C. difficile* amongst residents of RCFs in South Africa, but this is needed to guide recommendations for empiric antibiotic prescribing and infection control practices in these facilities. We performed a cross-sectional microbiological prevalence survey at three RCFs in Cape Town, South Africa, to determine the prevalence of colonization with ESBL-producing *Enterobacteriaceae* (ESBL-E), MRSA and toxigenic *C. difficile*; and identify risk factors for colonization.

## Methods

### Study setting and population

There are approximately 30 RCFs in the Cape Town metropolitan area. The majority of these institutions are operated by a non-profit organisation, the Cape Peninsula Organisation for the Aged (CPOA), which operate 25 facilities with ~ 3000 residents. We selected three facilities for inclusion in a cross-sectional prevalence survey. Facility selection was based on the following parameters: 1) a review of resident profiles (socioeconomic status, ethnicity) to approximate a broadly representative demographic sample of RCF residents; 2) availability of frail care facilities, which was not offered at all institutions; and 3) access to both public and private hospitals at different levels of care.

A random list of residents was generated at each facility, stratified by independent living and frail care areas. Frail care was defined as a specialised area in the RCF where residents require 24-h nursing care or supervision. These residents generally require assistance with activities of daily living (e.g. washing, dressing, eating), mobilisation, and taking of medicines [14]. Residents identified from the random lists were approached for participation in the study. In addition to active recruitment, information leaflets were distributed and formal presentations were done at each facility to encourage participation. Residents (or their legal representative where appropriate) expressing interest in participating were asked to provide written/telephonic informed consent prior to enrolment.

### Sources of data

*Risk factors for colonisation with MDROs and C. difficile.*

The following demographic and clinical data were collected at a single study visit through interviews and medical record reviews: presence of faecal/urinary incontinence, presence indwelling medical device, hospital exposure within last 6 months, systemic antibiotic exposure within the last 3 months, current use of proton pump inhibitors, functional and cognitive performance, presence of any skin ulceration, medical comorbidities (using the Charlson index), and any previous microbiological results in last 6 months. These were selected because of documented and putative associations with MDROs and *C. difficile* [4, 6, 9, 15–17]. Functional performance was assessed using the Katz Index of Independence in Activities of Daily Living (Katz ADL) which evaluates ability to perform ADLs and plan selfcare [18]. Scores ≤2 indicate severe functional impairment, 3–5 mild-to-moderate impairment, and 6 indicates independence. The presence of dementia was ascertained from medical records and through clinical assessment by the study doctor combined with simple screening tools (3-word recall) and the assessment of the facility nursing staff [19, 20]. All data were collected using standardised case report forms.

#### Microbiological data

Skin swabs of nasal, axillary and inguinal areas were performed to screen for carriage of MRSA. Stool was collected from each participant to screen for colonisation with ESBL-E and toxigenic *C. difficile*. All specimens were processed at the National Health Laboratory Services (NHLS) clinical microbiology laboratory at Groote Schuur Hospital, Cape Town. Skin swabs and stool samples were plated onto chromogenic screening agar, ChromID MRSA and ChromID ESBL agar plates (bioMérieux, Marcy I’Etoile, France). After incubation, suggestive colonies were identified and antibiotic susceptibility testing was performed using the Vitek 2 System (bioMérieux), and interpreted with Clinical Laboratory Standards Institute (CLSI) 2017 criteria. We did not screen for vancomycin-resistant Enterococci due to low prevalence in South African hospitals. Although carbapenem-resistant Enterobacteriaceae were not specifically screened for, these are also detected on the ChromID ESBL agar plates. An automated nucleic acid amplification test, Xpert *C. difficile* (Cepheid, Sunnyvale, CA, USA) was initially used to screen for toxigenic *C. difficile* in stool samples. This was later changed to a two-step algorithm where samples were screened with the dual antigen (glutamate dehydrogenase (GDH) and toxins A and B) with a C. Diff Quik Chek Complete test (TechLab, Blacksburg, VA, USA). *C. difficile* carriage was defined by positivity of both GDH and toxin assays; GDH-positive and toxin-negative samples reflexed to Xpert *C. difficile* testing.

### Analysis

The primary outcome measure was the proportion of residents colonised with MDROs and toxigenic *C. difficile*. Assuming a combined population of ~ 420 residents at the recruitment facilities, a sample size of 150 was planned to detect an ESBL-E colonisation prevalence of 20% with 5% precision. Associations between MDRO colonisation and participant characteristics were identified using the Wilcoxon rank sum test for continuous variables and χ^2^ test for categorical variables. Logistic regression was used to determine the risk factors associated with colonisation. Univariable analysis included the following pre-specified variables, plus significant associations identified in the descriptive analysis: hospitalisation and/or antibiotic exposure within the previous 3 or 6 months, non-ambulatory status, presence of pressure ulcers, and Charlson score. These variables were included in a multivariable model to adjust for potential confounding, using a backward stepwise selection strategy (*P* < 0.2). We combined significant predictors and evaluated accuracy for predicting MDRO colonisation by calculating the area under the receiver operating characteristic curve (AUROC). Analysis was performed in Stata (Version 14.2; Stata Corp, College Station, Texas, USA).

## Results

### Characteristics of study population

The combined population size of the three selected RCFs was 497, including 160 (32%) residents in frail care. Between March 2017 and April 2018, 172 (35%) residents were approached for participation: 18 declined and a total of 154 participants enrolled (Fig. S1 in supplementary material). The cohort included 59 (38%) residents from frail care and 95 from independent living areas. Median age was 79 years (interquartile range (IQR) 74–86) and 111 (72%) residents were female. Thirty-seven (24%) participants were bed- or chair-bound and the majority (*n* = 102, 67%) had Katz scores ≥5, indicating limited/no functional impairment. Forty-five (29.2%) had a diagnosis of dementia; median Charlson score was 1 (IQR 0–2). Urinary incontinence was present in 56 (36%) of participants and faecal incontinence in 24 (16%). Median time in the residence at the time of study participation was 41 months (IQR 17–72). Eighteen (12%) participants had been admitted to hospital in the previous six months and 38 (25%) had received systemic antibiotics in the previous three months.

### Prevalence of colonisation with MDROs and *C. difficile*

**Fig. 1:**
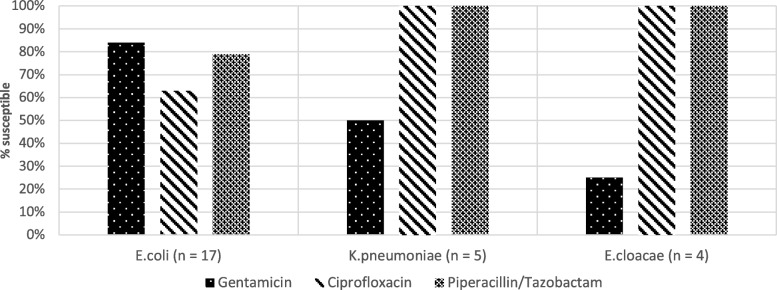
Susceptibility of ESBL-E isolates to commonly-used antibiotics

Stool samples were obtained from 119 residents. ESBL-E colonisation was detected in 27/119 (23%; 95% confidence interval (CI), 16–31%), comprising the following organisms: *E. coli* (17/27 isolates, 63%), *K. pneumoniae* (5/27 isolates, 19%), *E. cloacae* (4/27 isolates, 15%), and a single participant with mixed growth of *E. cloacae* and *E. coli*. Additional resistance to ciprofloxacin was detected in 19% (5/27), piperacillin-tazobactam in 11% (3/27) and gentamicin in 30% (8/27) (Fig. 1). All isolates were susceptible to carbapenems.

One hundred fifty-two sets of skin swabs were collected. A set was defined as three single swabs used to sample the nares, axillae and groin from an individual participant. MRSA was recovered from 13/152 (9%; 95% CI, 9–14%) individuals. The frequency of MRSA colonisation according to sampling site was: nasal 47%, groin 33% and axillae 20%. Four (3%, *n* = 117) participants had evidence of concurrent MRSA and ESBL-E colonisation.

Two (1.7%, *n* = 119) stool samples from asymptomatic residents were positive for *C. difficile;* both detected using the GDH antigen and toxin assay (*n* = 81). The remainder (*n* = 38) were tested using a nucleic acid amplification test with no positive results.

### Factors associated with MDRO colonisation

A significantly higher proportion of participants colonised with ESBL-E had urinary and/or faecal incontinence (59.3% vs. 33.7% in those not colonised; *P* = 0.02) (Table 1). The prevalence of ESBL-E amongst participants with incontinence was 34% (16 cases, *n* = 47), and a 2.9-fold increased odds (95% CI 1.2–6.9) of EBSL-E colonisation with any form of incontinence. ESBL-E colonisation was also associated with lower Katz ADL scores; there was a 1.3-fold (95% CI 1.0–1.6; *P* = 0.03) increased odds of colonisation for every 1-point reduction in the Katz ADL. Incontinence remained an independent predictor of ESBL-E colonisation on multivariable analysis, adjusted odds ratio (OR) 3.2 (1.3–8.1) (Table 2). ESBL-E colonisation was present in 53.3% (8 cases, *n* = 15) of participants with a combination of incontinence plus Katz score ≤ 2, significantly higher compared to participants without either condition (13.8%; 9 cases, *n* = 65). However, the discriminatory value of this risk factor combination was poor with AUROC 0.67 (95% CI 55–78). Colonized individuals were observed to have a higher median Charlson score (2 v 1, *p* = 0.06). There were no other associations between pre-specified risk factors and colonisation with ESBL-E (Table 1).

**Table 1:** Associations with ESBL-E colonisation

	Colonised (*n* = 27)	Not colonised (*n* = 92)	Prevalence ESBL-E (%)	*P*-value
Facility				
Facility 1	15 (55.6)	33 (35.9)	31.2	0.109
Facility 2	12 (44.4)	53 (57.6)	18.5	
Facility 3	0 (0)	6 (6.5)	0	
Time in facility, months	43.9 (22.9–65.2)	40.7 (14.3–73.6)	NA	0.992
Frail care resident	12 (44.4)	26 (28.3)	31.6	0.113
Any incontinence	16 (59.3)	31 (33.7)	34.0	0.017
Hospital exposure in last 6 months	10 (37.0)	21 (22.8)	32.3	0.139
Systemic antibiotic exposure last 3 months	8 (29.6)	18 (20.0)	30.8	0.291
Previous positive culture from a clinical specimen^a^	7 (36.8)^b^	20 (39.2)^c^	25.9	0.856
Bedbound or chair-bound	9 (33.3)	17 (18.5)	34.6	0.100
Katz score	6 (2–6)	6 (4–6)	NA	0.048
Dementia	10 (37.0)	20 (21.7)	33.3	0.107
Charlson index score	2, (1, 2)	1, (1, 2)	NA	0.058
Currently using PPI	8 (29.6)	19 (70.4)	19.6	0.090

Data are median (IQR) or n (percent). PPI, proton pump inhibitor

a. Includes microbiological evidence of *S. aureus*, Enterobacteriaceae, *C. difficile*

b*.n* = 19

c*.n* = 51

**Table 2:** Univariable and multivariable analysis of risk factors associated with ESBL-E colonisation

	Univariable		Multivariable (*n* = 117)	
Parameter	Odds ratio (95% CI)	*P* value	Odds ratio (95% CI)	*P* value
Any incontinence	2.9 (1.2–6.9)	0.019	3.2 (1.3–8.1)	0.013
Katz ADL	1.3 (1.0–1.6)	0.027		
Systemic antibiotic exposure last 3 months	1.7 (0.6–4.5)	0.294		
Hospital exposure in last 6 months	1.9 (0.8–4.9)	0.143	2.0 (0.8–5.5)	0. 154
Non-ambulatory	2.2 (0.8–5.7)	0.105		
Charlson score	1.4 (0.9–2.2)	0.119		

Katz ADL score, antibiotic exposure, non-ambulatory status, and Charlson score were dropped from the multivariable model due to P-value exceeding including pre-defined inclusion threshold (*P* < 0.2). Presence of pressure ulcers was not included as a predictor due to insufficient data (*n* = 4)

As shown in Table 3, participants colonised with MRSA had resided in their respective facilities for significantly less time compared to those who were not colonised with MRSA (20.9 vs 44.2 months; *P* = 0.04). There was a numerically higher proportion of MRSA-colonised individuals in frail care areas (61.5% vs. 36.0% in independent living areas; *P* = 0.07). The prevalence of MRSA colonisation amongst those in frail care was 13.8% (8 cases, *n* = 58), a non-significant 2.8-fold (95% CI, 0.9–9.2) increased odds of MRSA compared with participants residing in independent living areas. Multivariable analysis was not performed for MRSA colonisation because of low case numbers.

**Table 3:** Associations with MRSA colonisation

	Colonised (*n* = 13)	Not colonised (*n* = 139)	Prevalence of MRSA (%)	P-value
Facility				
Facility 1	6 (46.2)	55 (39.6)	9.8	0.167
Facility 2	5 (38.5)	78 (56.1)	6.0	
Facility 3	2 (15.4)	6 (4.3)	25.0	
Time in facility, months	20.9 (17.3–36.4)	44.2 (17.6–76.7)	NA	0.042
Frail care resident	8 (61.5)	50 (36.0)	13.0	0.070
Any incontinence	5 (38.5)	57 (41.0)	8.1	0.858
Hospital exposure in last 6 months	2 (15.4)	39 (28.1)	4.9	0.325
Systemic antibiotic exposure last 3 months	3 (25.0)	35 (25.6)	7.9	0.967
Previous positive culture from a clinical specimen^a^	4 (50)^b^	33 (40.2)^c^	10.8	0.592
Mobility status (bedbound/chair bound)	5 (38.5)	32 (23.0)	13.5	0.215
Katz score: median	5.5 (4–6)	6 (3–6)	NA	0.766
Dementia	4 (30.8)	41 (29.5)	8.9	0.923
Charlson index score	1 (0–2)	1 (0–2)	NA	0.848
Currently using PPI	3 (23.1)	10 (76.9)	8.1	0.701

Data are median (IQR) or n (percent). *PPI* Proton pump inhibitor

a. Includes microbiological evidence of *S. aureus*, Enterobacteriaceae, *C. difficile*

b*. n* = 8

c. *n* = 82

## Discussion

Determining the prevalence of colonisation with MDROs and *C. difficile* amongst RCF residents is important to inform empiric antibiotic selection and infection control practices. In South Africa, guidelines for managing RCF residents with infection are not based on local data, and this knowledge gap formed the rationale for the present study. We found that amongst 154 residents at three RCFs in Cape Town, the prevalence of ESBL-E and MRSA colonisation was 23 and 8%, respectively. *C. difficile* carriage was uncommon, identified in only two participants. Urinary or faecal incontinence and poor functional status were associated with ESBL-E carriage, and there was a trend towards increased risk of MRSA colonisation amongst residents in frail care.

There is a large amount of variability in published MDRO prevalence amongst long-term care facility residents. Estimates of ESBL-E colonisation in European series ranged between 4 and 64% [8, 9, 16, 21], similar to reports from the US [4, 15]. The wide range in prevalence is likely due to heterogeneity in study population. For example, inconsistent definitions of ‘long-term care facility’ are applied, some of which encompass acute care step down facilities expected to have higher prevalence of MDROs compared with RCFs, where residents are less sick and have less exposure to antibiotics [22–24]. ESBL-E colonisation was detected in 12% of residents (*n* = 119) in 3 residential aged care facilities in Australia [25]. Similar to our study the majority of residents were highly mobile and no association between recent antibiotic use, length of stay, urinary catheterisation, presence of diarrhoea and ESBL-E colonisation was found. The reported rates of *C. difficile* were also very low (1%), as in our study. In Belfast, Ireland, very high rates of ESBL-E colonisation (40%) were reported from 294 residents across 16 nursing homes; in contrast to our study, residents generally had high exposure to systemic antibiotic therapy, which was a significant risk factor for colonisation with ESBL-E [26].

These observations support our hypothesis that the local prevalence of colonisation in RCFs would be similar to that in high income settings. This high prevalence of ESBL-E colonisation (23%), plus additional resistance to ciprofloxacin (18%) amongst residents from RCFs in Cape Town suggests risk of treatment failure with the use of third generation cephalosporins and quinolones for common infection syndromes such as urinary tract infection and pneumonia.

Our findings are consistent with others showing Gram-negative bacteria to be the most prevalent multi-resistant pathogens recovered from RCF residents. For example, a cross-sectional study at a large LTCF in Boston found that 51% of sampled residents (*n* = 84) were colonised with multi-drug resistant Gram-negative bacteria compared to MRSA in 28% and vancomycin-resistant enterococci in 4% [4]. A longitudinal study conducted at a LTCF in Northern Ireland demonstrated similar results, with half of included residents (*n* = 64) positive for ESBL-E and a quarter for MRSA [16].

Poor functional status (i.e. residents requiring assistance with ADLs) and impaired mobility, with or without dementia, have been identified as significant factors for ESBL-E and MRSA colonisation [9]. In our study poor functional status (i.e those with a low Katz ADL score) and any form of incontinence were significantly associated with ESBL-E colonisation. The prevalence of ESBL-E colonisation with the combination of incontinence and Katz score ≤ 2 was high (53%), but had poor discriminatory value. Similar observations have been reported from high-income countries. In a study from Melbourne, Australia, where 115 residents from 4 facilities were screened, faecal incontinence and significant functional dependence (low Katz ADL score) were also shown to be major factors for colonisation with MDROs [27]. Similar predictors for MDR Gram-negative colonisation were found in a LTCF cohort in Boston: faecal incontinence, need for assistance with ADLs, advanced dementia and residing in units where more intensive nursing care was provided [4]. These factors may lead to higher levels of staff contact which result in cross-transmission [10]. It has been suggested that intensified infection prevention and control (IPC) measures, such as wearing of gowns and gloves by healthcare workers [28] and enhanced hygiene practices should be implemented for residents at high risk for MDRO colonisation [29]. Screening for ESBL-E and isolation of carriers outside of outbreak settings is controversial, and more evidence is required to understand the impact of this strategy to prevent transmission [30].

A comparatively low prevalence of MRSA colonisation (9%) was seen in our cohort, in contrast to studies in high income settings where MRSA prevalence ranged between 16 and 50% in various LTCF populations [31]. This discrepancy may be a consequence of circulating epidemic MRSA strains in the United States [34], which has not been the case in South Africa [35]. Shorter median time spent in RCFs was associated with MRSA colonisation in our study (20.9 versus 44.2 months for those not colonised). This may have been a chance finding due to low case numbers, and is susceptible to confounding factors which could not be adjusted for, such as visits to acute care facilities, which increases risk of MRSA acquisition [15], and differences in antibiotic therapy and IPC practices of attending physicians. There was a trend towards higher MRSA colonisation amongst residents in frail care; this has been observed in other settings and is possibly related to more frequent use of invasive medical devices, chronic wounds, and antibiotic exposure in this population [36].

CDI is endemic in RCFs in high income countries with incidence rates of 2.3 cases/10,000 resident days reported [37]. In contrast, only 2/119 (< 2%) samples were positive for *C. difficile* in our study. Studies at a Cape Town tertiary hospital found that 9–16% of acute diarrhoeal illnesses were associated with *C. difficile* infection, and the annual incidence of hospital-acquired diarrhoea was much lower compared to high income countries [38, 39]. These observations reflect the wide prevalence ranges for *C. difficile* which has a complex epidemiology across different settings, influenced by strain type, infection control and prescribing practices [40–42]. Active surveillance for carriers of toxigenic *C. difficile* has been advocated in high burden settings [43], but our findings suggest this may not be necessary in South African RCFs.

Our study has several limitations. As a result of limited resources we could not recruit residents from all RCFs in Cape Town, and selected a subset on the basis of representative demographics. Further limiting generalisability, we were unable to include all residents from the three participating facilities, and there were imbalances in number of participants across the RCFs. Although we generated randomised lists of residents at each facility, there is inherent bias in the recruitment process, and residents with MDRO colonisation may have been systematically excluded. We attempted to preferentially enrol residents in frail care areas in order to capture the highest risk group, but consent was more challenging in this population, skewing the sample towards independent living and less functional impairment. Our power to detect associations with MDRO colonisation was limited by low prevalence of MRSA colonisation, and because only 77% (119/154) of participants were willing to provide stool samples for ESBL-E screening. Although reliable systems were in place to collect clinical data, antibiotic exposure may have been underestimated as medications received during hospital admissions and clinic/general practitioner visits were incompletely documented. Finally, data collection occurred over a prolonged period due to logistic limitations and this may have influenced our results as colonisation prevalence is known to change over time [44].

## Conclusions

Notwithstanding these limitations, our survey demonstrated a relatively high prevalence of colonisation with MDROs, particularly ESBL-E, but low *C. difficile* carriage amongst residents of RCFs in Cape Town, South Africa. This has important implications for practice, including review of local antibiotic prescribing guidelines to ensure appropriate initial therapy for RCF residents. Crucially, IPC interventions such as improved healthcare worker hand hygiene and barrier nursing, as well as antibiotic stewardship, should be implemented, and possibly targeted at higher risk residents, including those with incontinence and lower functional status, to interrupt the transmission of MDROs in RCFs.

## Data Availability

The datasets used and/or analysed during the current study are available from the corresponding author on reasonable request.
